# Association of neutrophil to high-density lipoprotein cholesterol ratio with overactive bladder: a population-based study

**DOI:** 10.3389/fendo.2025.1541294

**Published:** 2025-05-22

**Authors:** Huifeng Zhang, Yaying Xu, Lele Chen

**Affiliations:** ^1^ Department of Cardiovascular, The First Affiliated Hospital, and College of Clinical Medicine of Henan University of Science and Technology, Luoyang, China; ^2^ Department of Endocrinology, The First Affiliated Hospital, and College of Clinical Medicine of Henan University of Science and Technology, Luoyang, China; ^3^ Department of Vascular Surgery, Southeast Yu Branch of Henan Provincial People’s Hospital, Zhumadian, China

**Keywords:** overactive bladder, neutrophil to high-density lipoprotein cholesterol ratio, inflammation, NHANES, cross-sectional study

## Abstract

**Background:**

Inflammation is involved in the progression of overactive bladder (OAB). This study aimed to investigate the association between the neutrophil to high-density lipoprotein cholesterol ratio (NHR) and OAB.

**Methods:**

Data from seven cycles of the National Health and Nutrition Examination Survey (NHANES) from 2005 to 2018 were analyzed to examine the relationship between NHR and OAB. Multivariable logistic regression model was used to estimate the association between NHR and OAB, along with restricted cubic spline regression to assess the dose-response relationship. Additionally, subgroup analyses were conducted.

**Results:**

Among 29,315 participants, 5,815 had OAB. After covariate adjustment, we found that NHR was positively associated with the odds of OAB. Notably, the relationship between NHR and OAB was nonlinear. There was a threshold at NHR = 2.85. When NHR < 2.85, no significant association was observed; when NHR ≥ 2.85, a positive association emerged. Subgroup and interaction analyses further revealed that the link between NHR and OAB was stronger in obese participants.

**Conclusion:**

Our results indicate a nonlinear association between NHR and OAB. When NHR is ≥ 2.85, there is a strong positive association between NHR and the odds of OAB. The interaction between obesity and NHR further increases the prevalence of OAB. Prospective and multicenter studies are needed in the future to elucidate the potential mechanism underlying the association between NHR and OAB and determine the causal relationship.

## 1 Introduction

Overactive bladder (OAB) is a common urinary system disease characterized by symptoms such as urgency, frequent urination, and nocturia, which seriously affects the quality of life of millions of patients worldwide (1, 2). Earlier, it was estimated that in the United States, the prevalence of OAB exceeded 16% for both men and women (3). Recent studies have shown that the overall prevalence of OAB in American men has increased significantly from 2005 to 2020 (4). OAB not only leads to significant psychological stress and social isolation but also reduces work efficiency and imposes a considerable burden on the medical system (5). The etiology of OAB is complex and involves neural factors, smooth muscle factors, and the influence of lifestyle and environment. With the deepening of research, the potential association between systemic inflammation and OAB has received increasing attention (6).

Recent studies have shown that chronic inflammation may play a role in the pathogenesis of OAB (4, 7). The neutrophil-to-high-density lipoprotein cholesterol ratio (NHR) is an emerging inflammatory marker that reflects the balance between neutrophil activation and the protective effect of high-density lipoprotein (HDL) (8, 9). An increase in neutrophil count is associated with a systemic inflammatory response, while HDL is widely recognized for its anti-inflammatory properties (10). Extensive research has shown that NHR has the potential to predict multiple diseases, such as depression, chronic kidney disease, metabolic dysfunction-associated steatotic liver disease (MASLD), periodontitis, etc. (11–14).

Increasing NHR indicates an intensified inflammatory state, which may aggravate bladder dysfunction and promote the occurrence of OAB. More and more evidence shows that inflammatory cytokines can affect the function of bladder smooth muscle and detrusor overactivity, revealing that NHR may affect bladder health through this pathway (15, 16). However, the relationship between NHR and OAB has not been fully studied. Understanding this relationship may provide new therapeutic targets or prevention strategies for managing OAB.

In this study, we hypothesized that an increase in NHR increases the prevalence of OAB. We evaluated this conjecture by analyzing real-world clinical samples from the National Health and Nutrition Examination Survey (NHANES).

## 2 Methods

### 2.1 Study population

NHANES is an ongoing national cross-sectional study conducted by the Centers for Disease Control and Prevention of the United States every two years since 1999, targeting non-institutionalized civilian residents of the United States. NHANES uses a stratified multi-stage probability sampling method to obtain a representative sample and obtains basic demographic, socioeconomic information, health-related, and nutritional status of participants through personal interviews, standardized questionnaires, and physical examinations. The Ethics Review Committee of the National Center for Health Statistics has approved all NHANES protocols, and each participant has provided written informed consent. In this study, the data are from seven non-repetitive cycles (2005 - 2018) of NHANES. Detailed information and data about NHANES can be found online at https://www.cdc.gov/nchs/nhanes/index.htm.

**Figure 1:**
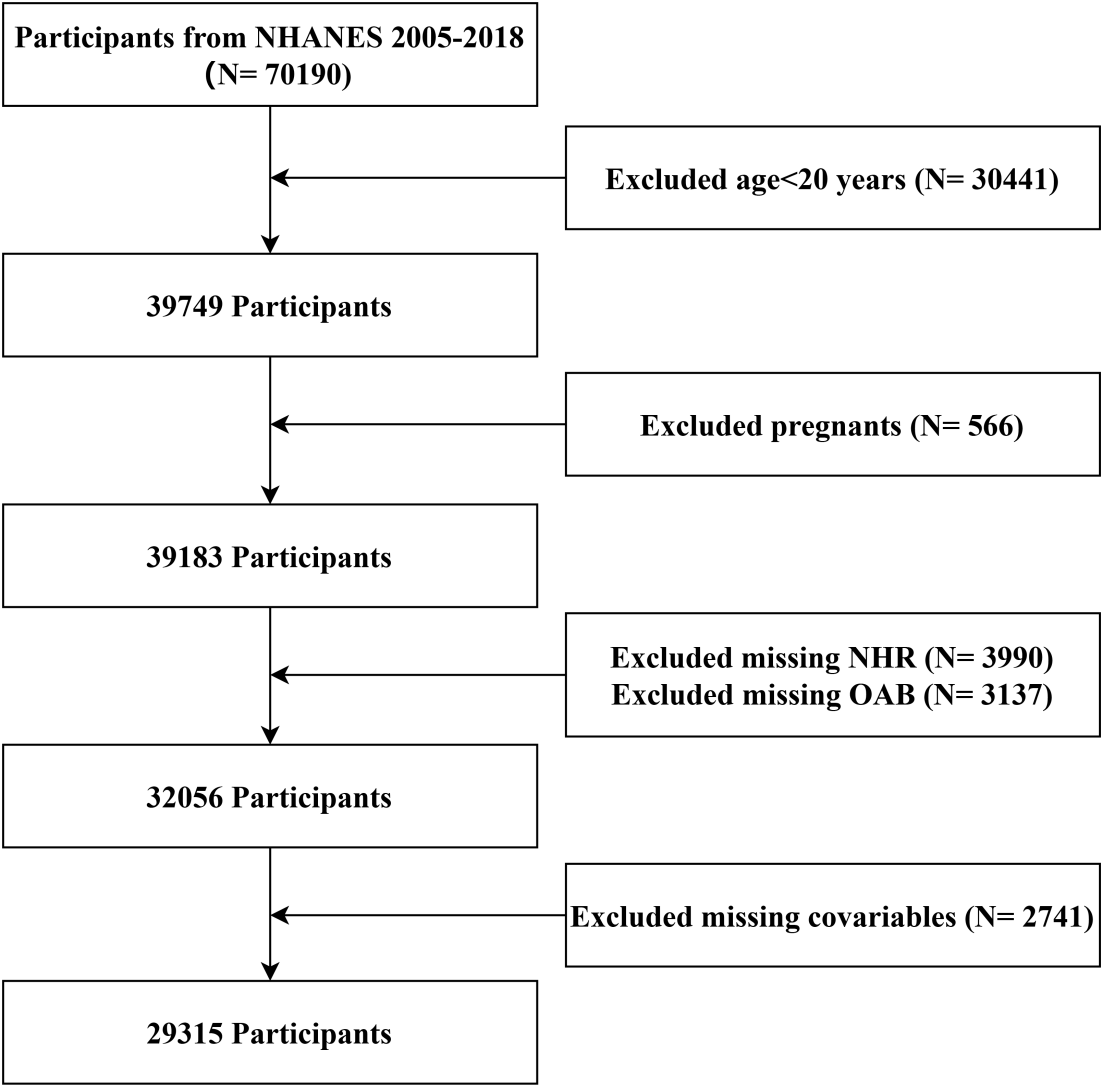
Flowchart of participant selection.

The process of inclusion and exclusion is shown in **Figure 1**. Initially, 70,190 participants were included in this study. First, according to the definition of the age range of adults in previous studies, we excluded individuals younger than 20 years old (n = 30,441); participants who were pregnant (n = 566), had missing values for NHR (n = 3,990), OAB (n = 3,137), and necessary covariates (n = 2,741) were also excluded. Finally, 29,315 subjects were included in this study, representing 180 million non-institutionalized American people after weighting.

### 2.2 Measurement of NHR

NHR is the ratio of neutrophil count (10^^3^ cells/μL) to high-density lipoprotein cholesterol (HDL-C) (mmol/L) (17). Both the numerator and denominator of NHR are obtained through laboratory tests. Blood samples are collected by established venipuncture protocols and procedures. The absolute number of neutrophils is measured using the UniCel DxH 800 analyzer. HDL-C is measured on Roche Modular P and Roche Cobas 6000 chemistry analyzers.

### 2.3 Definition of OAB

According to the International Continence Society, the primary characteristics of overactive bladder (OAB) are urinary urgency, incontinence (UUI), and nocturia, and assessing these features is essential for diagnosing OAB. The survey was administered by trained researchers utilizing questionnaires. To evaluate the severity of UUI, participants answered two key questions: “Have you experienced urine leakage or loss of control due to an urgent need to urinate in the past 12 months, making it difficult to reach the toilet in time?” and “How often does this happen?” (5). For assessing nocturia, another question posed was: “In the past 30 days, how many times did you typically get up at night to urinate from the time you went to bed until you got up in the morning?” (18). After gathering the responses, the researchers applied the Overactive Bladder Symptom Score (OABSS) to determine the severity of OAB (19, 20). Participants scoring 3 or more were categorized as having OAB (21–23). For additional details, please refer to **Supplementary Figure 1**. It is particularly important to note that relying solely on questionnaire-based diagnosis of OAB is a significant limitation.

### 2.4 Definition of covariates

All covariates include demographic, socioeconomic, lifestyle, and health-related characteristics. Demographic and socioeconomic variables were collected: sex (male/female), age (continuous), race (non-Hispanic white, non-Hispanic black, Mexican American, other Hispanic, and multiracial), educational background (college degree or above and others), marital status (married and others), and poverty income ratio (PIR). The PIR is calculated based on the poverty guidelines of the U.S. Department of Health and Human Services (website: http://aspe.hhs.gov/poverty/13poverty.cfm). According to the official recommendations of the NHANES, PIR can be classified as follows according to the eligibility of the Supplemental Nutrition Assistance Program (SNAP): 0.00–1.30, >1.30–3.50, and >3.50 and above (24, 25). Lifestyle and health-related variables were collected: smoking status (current smoker and others), drinking status (current drinker and others), physical activity (inactive/moderate/active/unknown), dietary health (Healthy Eating Index-2015), and body mass index (BMI). It should be noted that according to the definition of the World Health Organization (WHO), BMI is classified into the following categories: < 25 kg/m², 25–30 kg/m², > 30 kg/m² (26, 27). In addition, health-related characteristics were considered: cancer (no/yes), diabetes (no/yes/borderline), hypertension (no/yes), chronic kidney disease (CKD) (no/yes), cardiovascular disease (CVD) (no/yes), liver disease (no/yes), hyperlipidemia (no/yes), and use of lipid-lowering drugs (no/yes).

### 2.5 Statistical analysis

Given the complex multistage sampling design of the National Health and Nutrition Examination Survey (NHANES), appropriate sample weights were used. Continuous variables are expressed as weighted mean ± standard error (SE), while categorical variables are expressed as count (weighted percentage, %). The chi-square test is used to compare differences between groups for categorical variables, and the t-test is used to compare differences between groups for continuous variables. Age-standardized prevalence estimates and 95% confidence intervals (CI) of OAB for quartile groups of NHR were calculated. Multivariable logistic regression models were used to estimate adjusted odds ratios (OR) and 95%CI to analyze the association between NHR and OAB. We used three different models: the crude model (Model 0) does not adjust for any variables. Model 1 adjusts for age, sex, race/ethnicity, marital status, PIR, educational level, drinking status, smoking status, physical activity, healthy diet, and BMI. Model 2 further adjusts for cancer, diabetes, hypertension, chronic kidney disease, cardiovascular disease, liver disease, hyperlipidemia, and use of lipid-lowering drugs based on Model 1. Stratified analysis was performed to evaluate the effect of NHR on the prevalence of OAB in different subgroups of demographic, socioeconomic, lifestyle, and health-related characteristics. In addition, the dose-response relationship between NHR and OAB was estimated by the restricted cubic spline (RCS) regression model. Finally, in the sensitivity analysis, we included the samples from the period of the major epidemic (2019-2020) in this study and conducted the analysis again.

All statistical analyses were performed using the R statistical programming language (64-bit version 4.3.1; R Foundation for Statistical Computing).

## 3 Results

### 3.1 Baseline characteristics

29,315 participants over 20 years old were included, representing 180 million non-institutionalized American adults (**Figure 1**). Among the 29,315 participants, 5,815 cases of OAB were identified. **Table 1** shows the baseline characteristics of the participants divided according to whether they have OAB. In general, the weighted average age of all participants was 47.24 ± 0.25 years, and 50.50% (n = 14,622) were female. More than two-thirds of the participants were non-Hispanic whites, 61.69% were well-educated, 64.18% were married or cohabiting, and 41.73% had a PIR > 3.5. Compared with the non-OAB group, OAB patients were more likely to be female, older, non-Hispanic black, with low educational attainment, unmarried, and have low income; in addition, OAB participants were less likely to currently drink alcohol and have active physical activity. They were more likely to be obese, to have cancer, diabetes, CVD, hypertension, CKD, liver problems, and hyperlipidemia, and more likely to use lipid-lowering drugs.

**Table 1:** Weighted characteristics of the study population(n=29,315).

Variable		OAB		P-value
	Total [n=29315]	No [n=23500]	Yes [n=5815]	
NHR	3.45 [0.03]	3.42 [0.03]	3.61 [0.04]	< 0.0001
Age	47.42 [0.25]	45.47 [0.24]	58.09 [0.35]	< 0.0001
Healthy Eating Index 2015	50.72 [0.20]	50.70 [0.20]	50.81 [0.31]	0.72
Sex				< 0.0001
Female	14622 [50.50]	11212 [48.44]	3410 [61.81]	
Male	14693 [49.50]	12288 [51.56]	2405 [38.19]	
Race				< 0.0001
Mexican American	4650 [8.31]	3779 [8.49]	871 [7.32]	
Non-Hispanic Black	6015 [10.31]	4404 [9.26]	1611 [16.08]	
Non-Hispanic White	12959 [69.26]	10547 [69.83]	2412 [66.18]	
Other Hispanic	2802 [5.29]	2222 [5.29]	580 [5.34]	
Other Race	2889 [6.82]	2548 [7.14]	341 [5.09]	
Education level				< 0.0001
Less than some college or AA degree	13706 [38.31]	10343 [36.21]	3363 [49.85]	
Some college or AA degree	15609 [61.69]	13157 [63.79]	2452 [50.1]	
Marital status				< 0.0001
Other	11684 [35.82]	8984 [34.97]	2700 [40.50]	
Married or living with partner	17631 [64.18]	14516 [65.03]	3115 [59.50]	
Income to poverty ratio				< 0.0001
<1.3	8145 [18.70]	6122 [17.42]	2023 [25.72]	
1.3-3.5	10243 [33.34]	8116 [32.65]	2127 [37.13]	
>3.5	8633 [41.73]	7472 [43.97]	1161 [29.49]	
missing	2294 [6.22]	1790 [5.96]	504 [7.66]	
Drinking				< 0.0001
No	8828 [23.84]	6443 [21.73]	2385 [35.38]	
Yes	20487 [76.16]	17057 [78.27]	3430 [64.62]	
Smoking				0.1
No	23275 [80.02]	18676 [80.23]	4599 [78.86]	
Yes	6040 [19.98]	4824 [19.77]	1216 [21.14]	
Physical activity				< 0.0001
Inactive	5676 [19.75]	4537 [19.72]	1139 [19.89]	
Moderate	6514 [24.24]	5401 [24.99]	1113 [20.13]	
Active	9775 [35.51]	8322 [37.04]	1453 [27.12]	
Missing	7350 [20.50]	5240 [18.25]	2110 [32.85]	
BMI (KG/M^2^)				< 0.0001
<25	8328 [29.79]	7150 [31.44]	1178 [20.78]	
25-30	9749 [32.96]	8044 [33.49]	1705 [30.07]	
>30	11238 [37.25]	8306 [35.08]	2932 [49.15]	
Cancer				< 0.0001
No	26576 [90.07]	21668 [91.47]	4908 [82.44]	
Yes	2739 [9.93]	1832 [8.53]	907 [17.56]	
DM				< 0.0001
No	21312 [77.59]	17997 [80.39]	3315 [62.26]	
Yes	5425 [13.80]	3503 [11.36]	1922 [27.17]	
Borderline	2578 [8.61]	2000 [8.25]	578 [10.57]	
CVD				< 0.0001
No	26166 [91.60]	21619 [93.58]	4547 [80.75]	
Yes	3149 [8.40]	1881 [6.42]	1268 [19.25]	
Hypertension				< 0.0001
No	16874 [62.36]	14761 [66.22]	2113 [41.26]	
Yes	12441 [37.64]	8739 [33.78]	3702 [58.74]	
CKD				< 0.0001
No	24114 [85.98]	20134 [88.34]	3980 [73.02]	
Yes	5201 [14.02]	3366 [11.66]	1835 [26.98]	
Liver disease				< 0.0001
No	28146 [96.46]	22679 [96.84]	5467 [94.39]	
Yes	1169 [3.54]	821 [3.16]	348 [5.61]	
Lipid - lowering drugs				< 0.0001
No	23763 [83.09]	19827 [85.52]	3936 [69.80]	
Yes	5552 [16.91]	3673 [14.48]	1879 [30.20]	
Hyperlipidemia				< 0.0001
No	8284 [29.35]	7144 [31.10]	1140 [19.79]	
Yes	21031 [70.65]	16356 [68.90]	4675 [80.21]	

Data were presented as number [weighted percentages] or means ± standard error. OAB overactive bladder, NHR neutrophil to high-density lipoprotein cholesterol ratio, DM diabetes, BMI body mass index, Kg kilogram, M^2^ square of meters, CVD cardiovascular diseases, CKD chronic kidney disease, OR odds ratio, CI confidence interval.

### 3.2 Independent association of NHR with OAB

**Figure 2:**
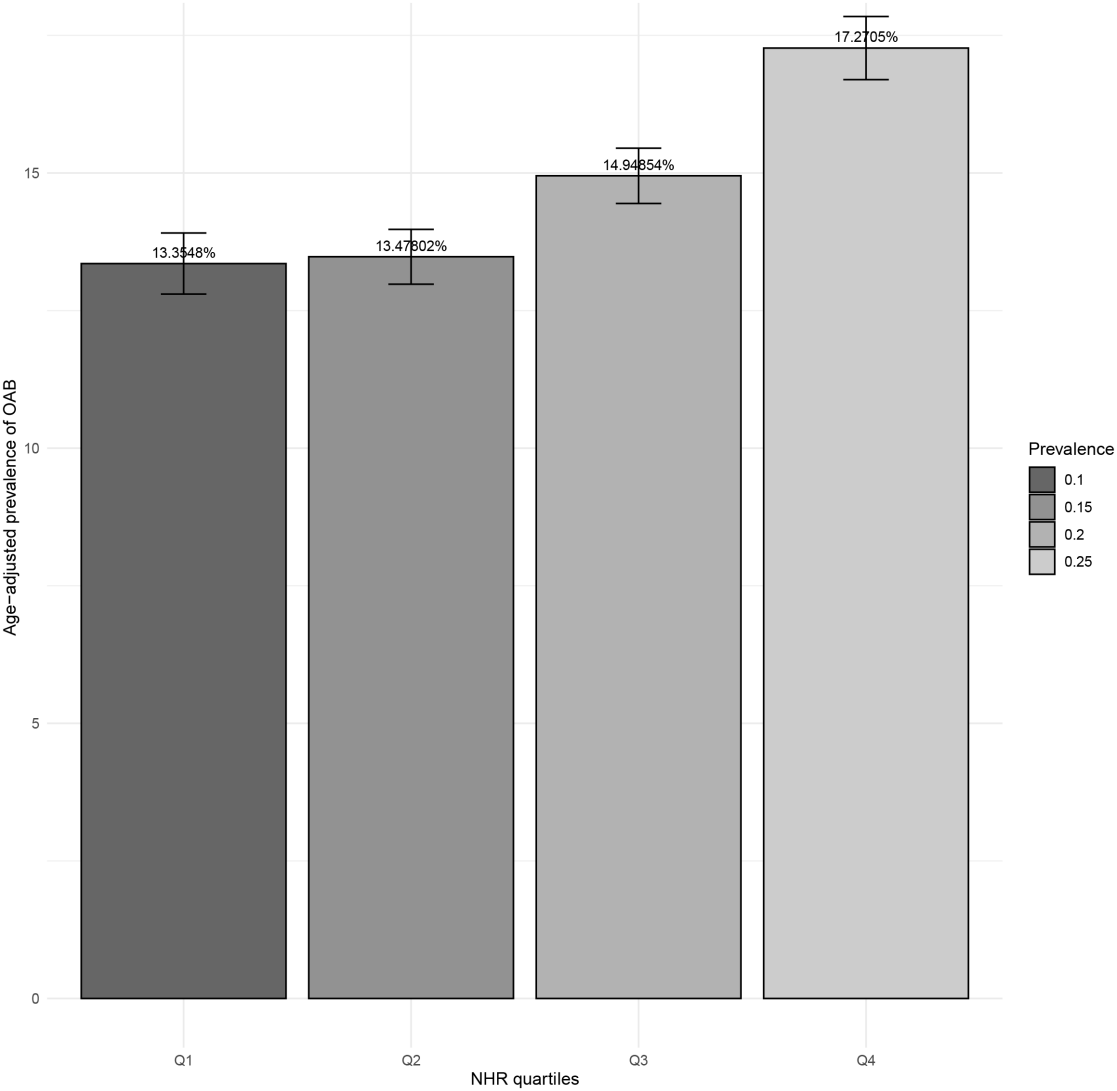
Age-adjusted prevalence of OAB in diferent levels of NHR. Numbers at the top of the bars represent the weighted percentage. Bar whiskers represent the 95% confidence level. OAB, Overactive bladder; NHR, neutrophil to high-density lipoprotein cholesterol ratio.

**Figure 2** shows the age-standardized prevalence of OAB in the quartiles of NHR. The prevalence of OAB demonstrated a gradual ascent among the four quartile groups of NHR. Specifically, the prevalence of OAB in the lowest quartile (Q1) of NHR was 13.35%, that in Q2 was 13.48%, in Q3 it was 14.95%, and in the highest quartile (Q4), the prevalence reached 17.27%.

**Table 2** shows the results of weighted logistic regression of NHR and OAB. In the crude model (Model 0), NHR was significantly associated with OAB [odds ratio (OR)=1.05, 95%CI: (1.03, 1.07), P<0.0001]. After adjusting for demographic factors, socioeconomic factors, lifestyle, and BMI (Model 1), the association between NHR and OAB was stronger [adjusted odds ratio (AOR)=1.06, 95%CI: (1.03, 1.08), P<0.0001]. After fully adjusting for demographic factors, socioeconomic factors, lifestyle, BMI, lipid-lowering drugs, and health comorbidities (Model 2), the association between NHR and OAB was weakened but still significant [AOR=1.04, 95%CI: (1.01, 1.07), P=0.004]. To better reveal the changing trend of the prevalence of OAB with NHR, NHR was divided into four groups. In the original model (Model 0), compared with the lowest quartile group (Q1), the prevalence of OAB in Q2 [AOR: 1.03, 95% CI: (0.91, 1.17), P = 0.62] and Q3 [AOR: 1.08, 95% CI: (0.97, 1.21), P = 0.16] did not change significantly, while the prevalence in Q4 was significantly higher [AOR: 1.23, 95% CI: (1.11, 1.35), P < 0.0001]. In the partially adjusted Model 1, compared with the Q1 group, the prevalence of OAB in Q2 [AOR: 1.02, 95% CI: (0.89, 1.17), P = 0.75] and Q3 [AOR: 1.09, 95% CI: (0.96, 1.24), P = 0.16] did not change significantly, while the prevalence in Q4 was significantly higher [AOR: 1.26, 95% CI: (1.12, 1.42), P < 0.001]. The same was true in the fully adjusted Model 2. Compared with the Q1 group, the prevalence of OAB in Q2 [AOR: 1.05, 95% CI: (0.92, 1.19), P = 0.50] and Q3 [AOR: 1.07, 95% CI: (0.94, 1.21), P = 0.33] still did not change significantly, while the prevalence in Q4 was significantly higher [AOR: 1.18, 95% CI: (1.04, 1.34), P = 0.01].

**Table 2:** Association between NHR and OAB.

NHR	Model 0		Model 1		Model 2	
	OR [95%CI]	P-value	OR [95%CI]	P-value	OR [95%CI]	P-value
Per unit	1.05 [1.03,1.07]	<0.0001	1.06 [1.03,1.08]	<0.0001	1.04 [1.01,1.07]	0.004
Quartile 1	ref		ref		ref	
Quartile 2	1.03 [0.91,1.17]	0.62	1.02 [0.89,1.17]	0.75	1.05 [0.92,1.19]	0.50
Quartile 3	1.08 [0.97,1.21]	0.16	1.09 [0.96,1.24]	0.16	1.07 [0.94,1.21]	0.33
Quartile 4	1.23 [1.11,1.35]	<0.0001	1.26 [1.12,1.42]	<0.001	1.18 [1.04,1.34]	0.01
*P* trend		<0.0001		<0.001		0.01

Model 0 did not adjust for any confounding factors; Model 1: Adjust for age, sex, race/ethnicity, marital status, poverty income ratio, educational level, drinking status, smoking status, healthy diet, physical activity, and body mass index. Model 2 further adjusts for cancer, diabetes, hypertension, chronic kidney disease, cardiovascular disease, liver disease, hyperlipidemia, and use of lipid-lowering drugs based on Model 1. OR odds ratio, CI confidence interval, OAB overactive bladder, NHR neutrophil to high-density lipoprotein cholesterol ratio.

### 3.3 Dose-response effect between NHR and OAB

**Figure 3:**
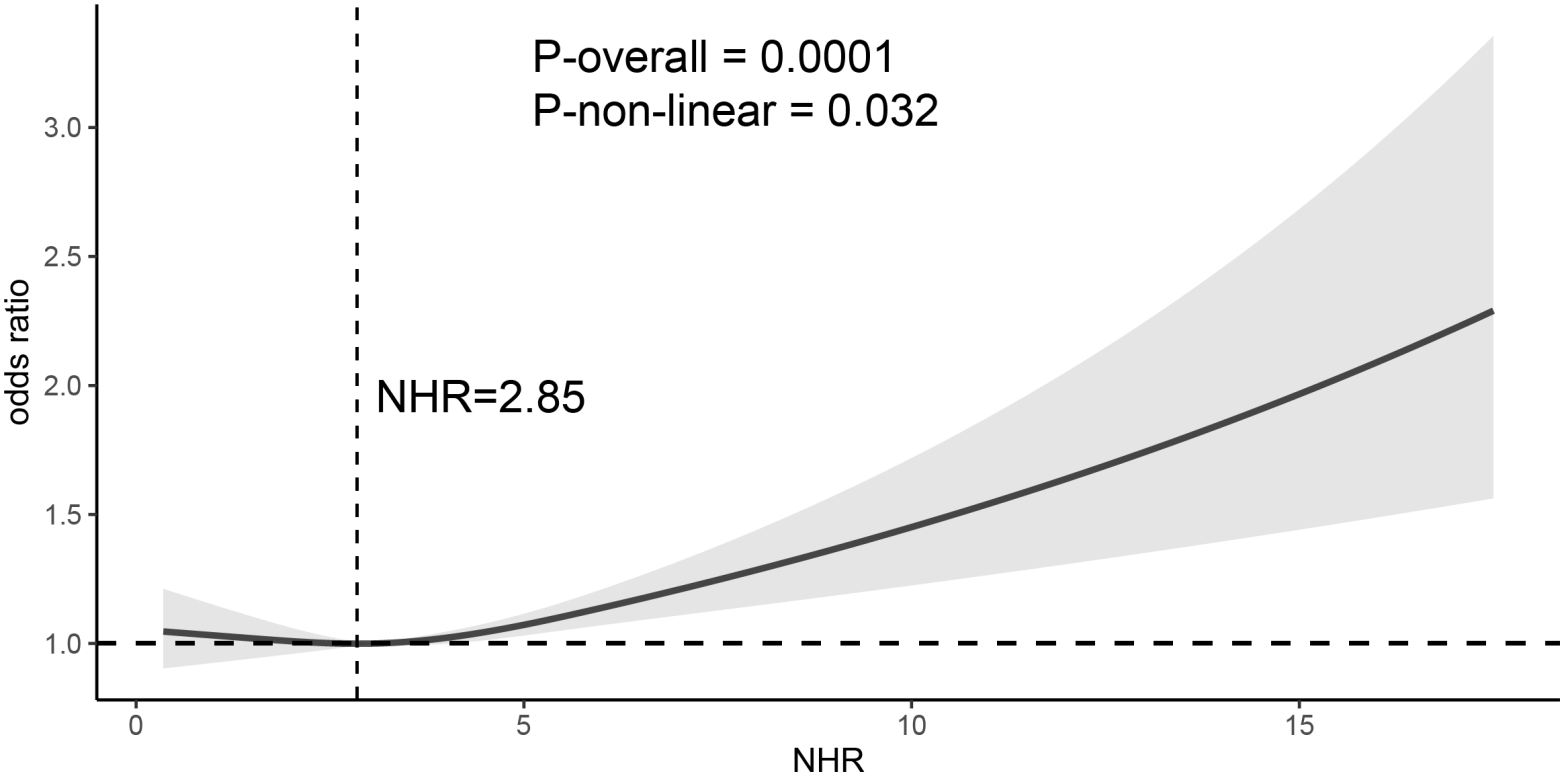
Nonlinear analysis of the association between NHR and OAB. In the restricted cubic spline analysis, it was adjusted for sex, age, race, educational level, partner status, poverty income ratio, smoking, alcohol consumption, healthy diet, weekly exercise level, dietary health, body mass index, hypertension, cardiovascular disease, chronic kidney disease, liver disease, cancer, diabetes, hyperlipidemia, and lipid - lowering drugs. NHR, neutrophil to high-density lipoprotein cholesterol ratio; OAB, Overactive bladder.

To better understand the changing trend of OAB prevalence as NHR increases, RCS regression analysis was performed. As shown in **Figure 3**, after fixing the median value of NHR as the reference point, the curve showed a significant nonlinearity (P non-linear = 0.032). The NHR corresponding to the lowest point of OAB prevalence was 2.85. Further, a piecewise logistic regression was performed with NHR = 2.85 as the inflection point. As shown in **Table 3**, in the section where NHR < 2.85, all three models suggested that NHR was not associated with OAB (all P > 0.05); in the section where NHR ≥ 2.85, all three models suggested a positive association between NHR and OAB. The fully adjusted model (Model 3) suggested that for per 1-unit increase in NHR, the prevalence of OAB increased by 5% [AOR: 1.05, 95%CI: (1.01, 1.09), P = 0.01].

**Table 3:** Association between NHR and OAB through segmented NHR.

NHR	Model 0		Model 1		Model 2	
	OR [95%CI]	P-value	OR [95%CI]	P-value	OR [95%CI]	P-value
*<2.85*	0.92 [0.82,1.03]	0.14	0.91 [0.79,1.04]	0.17	0.89 [0.78,1.02]	0.10
*≥2.85*	1.06 [1.03,1.09]	<0.001	1.07 [1.03,1.11]	<0.001	1.05 [1.01,1.09]	0.01

Model 0 did not adjust for any confounding factors; Model 1: Adjust for age, sex, race/ethnicity, marital status, poverty income ratio, educational level, drinking status, smoking status, healthy diet, physical activity, and body mass index. Model 2 further adjusts for cancer, diabetes, hypertension, chronic kidney disease, cardiovascular disease, liver disease, hyperlipidemia, and use of lipid-lowering drugs based on Model 1. OAB, overactive bladder; NHR, neutrophil to high-density lipoprotein cholesterol ratio; OR, odds ratio; CI, confidence interval.

### 3.4 Subgroup analysis

**Figure 4:**
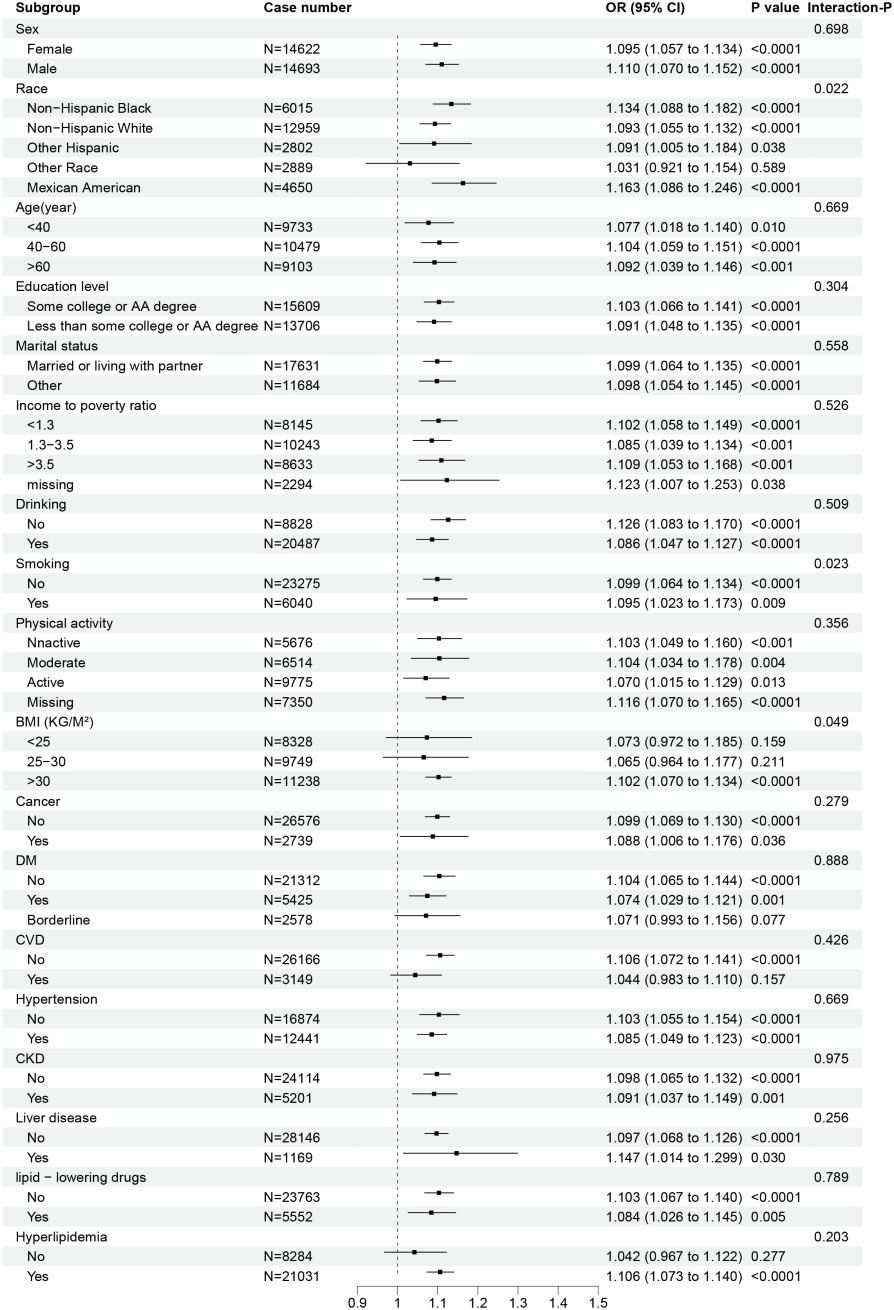
Subgroup analysis and multiplication interaction tests of the association between NHR and OAB. In subgroup analysis, it was adjusted for sex, age, race, educational level, partner status, poverty income ratio, smoking, alcohol consumption, weekly exercise level, dietary health, body mass index, hypertension, cardiovascular disease, chronic kidney disease, liver disease, cancer, diabetes, hyperlipidemia, and lipid - lowering drugs. NHR, neutrophil to high-density lipoprotein cholesterol ratio; DM, diabetes; BMI, body mass index; Kg, kilogram; M^2^, square of meters; CVD, cardiovascular diseases; CKD, chronic kidney disease; OR, odds ratio; CI, confidence interval.

As shown in **Figure 4**, in the subgroup analysis, there was a significant multiplicative interaction effect between BMI and NHR (P for interaction = 0.049). Specifically, in the obese subgroup (BMI > 30 kg/m^2^), the association between NHR and OAB was the strongest [AOR: 1.102, 95%CI: (1.070, 1.134), P < 0.0001]; in the overweight subgroup (30 kg/m^2^ > BMI > 25 kg/m^2^), there was no significant association between NHR and OAB [AOR: 1.065, 95%CI: (0.964, 1.177), P = 0.211]; in the subgroup with BMI < 25 kg/m^2^, NHR also did not affect the prevalence of OAB [AOR: 1.073, 95%CI: (0.972, 1.185), P = 0.159]. There was no significant interaction between BMI and NHR in other subgroups.

### 3.5 Sensitivity analysis

In the sensitivity analysis, after we included the samples from the period of the epidemic outbreak in 2019–2020 in the analysis, there were a total of 31,489 samples. The results of the weighted logistic regression were basically consistent with the above-mentioned results (**Supplementary Tables 1, 2**), further supporting our findings.

## 4 Discussion

This study analyzed the relationship between NHR and OAB by analyzing real-world sample data from the NHANES database. A total of 29,315 participants were included in this study, and approximately 180 million Americans over 20 years old were represented by weighting. Our results show that although NHR is independently positively associated with OAB in the overall population; however, the association between NHR and OAB is nonlinear. The inflection point of the association between NHR and OAB was determined to be at NHR = 2.85 through the dose-response relationship curve; below this value, there is no significant association between NHR and OAB. However, when NHR *≥* 2.85, for per 1-unit increase in NHR, the prevalence of OAB increases by 5%. In addition, a stronger association between NHR and OAB was observed in obese (BMI > 30 kg/m^2^) participants.

Although there is currently no research on the relationship between NHR and OAB, existing studies have shown that inflammation is related to the onset and symptom severity of OAB. A study consistent with ours reported the association between systemic immunity-inflammation index (SII) and OAB. Although the dose-response relationship was not explored, their study showed that compared with the lowest quartile group of SII, the prevalence of OAB in the participants in the second and third quartile groups did not change significantly, while the prevalence of OAB in the highest quartile group increased (4). A mediation analysis showed that white blood cell count (WBC) and neutrophil count partially mediate the association between diabetes-related markers (glycated hemoglobin, fasting blood glucose, and insulin) and OAB (7). This at least suggests that in the diabetic population, neutrophils may mediate the progression of OAB. Earlier, an epidemiological study using a multi-stage stratified cluster design investigated 5,503 adults in Boston. They found a positive association between elevated C-reactive protein (CRP) levels and OAB in both men and women (28). In addition, there is more direct evidence that a long-term inflammatory state leads to weakened and hypersensitive bladder function, and then leads to OAB-related symptoms (2, 29). Tyagi et al. (30) reported a significant increase in the concentration of multiple inflammation-related proteins in the urine of OAB patients, especially the levels of monocyte chemoattractant protein-1 (MCP-1) and soluble CD40 ligand (sCD40L) increased more than tenfold. Liu et al. (31) found a large number of mast cell infiltrations in the bladder wall of OAB patients. On the other hand, HDL-C has an anti-inflammatory effect. Uzun et al. (32) included 122 OAB patients and 62 age-matched controls without OAB. They found that HDL-C was significantly reduced in female OAB patients. Yuksel et al. (33) included 181 participants and found that those with lower HDL-C levels had a higher incidence of OAB. Baytaroglu et al. (34) suggested that higher low-density lipoprotein (LDL) levels are an independent predictor of OAB. As the ratio of neutrophil count to HDL-C, NHR can reflect the inflammation level in organisms. Therefore, NHR may be a good tool to help distinguish OAB and evaluate treatment effects.

This study also revealed that after dividing all populations into normal, overweight, and obese according to BMI, the association between NHR and OAB was only significant in the obese population, and there was a potential multiplicative interaction between BMI and NHR. Obese people usually have multiple physiological changes that may affect lower urinary tract function. Previous studies have confirmed that obesity indicators, including BMI, weight-adjusted waist index (WWI), body roundness index (BRI), waist circumference, etc., are related to the prevalence of OAB (20, 32, 33, 35, 36). Abdominal fat accumulation can increase abdominal pressure and exert continuous pressure on the bladder, which may lead to abnormal bladder function and increase the risk of OAB (37). In addition, the neuroendocrine disorders caused by leptin secreted by visceral adipose tissue and inflammatory cytokines can lead to enhanced noradrenergic sympathetic nerve activity and stimulation of the urothelium (38). As an inflammatory indicator, NHR may be higher in an obese state, because some studies have suggested that the inflammatory marker NHR successfully distinguishes obese individuals from normal individuals (39).

In terms of the mechanism, from the perspective of inflammation, neutrophils, as an important component of the immune system, play a crucial role in the inflammatory response. OAB may be accompanied by a local inflammatory state. For example, chronic low-grade bacterial bladder colonization may trigger a local inflammatory response in the bladder, which in turn exacerbates the symptoms of OAB (40). The increase in systemic neutrophils may be a systemic immune response of the body to local bladder inflammation. When there is local inflammatory stimulation in the bladder, the immune system is activated, prompting the bone marrow to produce and release more neutrophils into the bloodstream to deal with potential infections or inflammatory injuries (41). Therefore, the increase in systemic neutrophil levels may indirectly reflect the local inflammatory process in the bladder, and the neutrophil count component in NHR is thus associated with the bladder-related processes. Secondly, HDL-C has various physiological functions such as anti-inflammation and anti-oxidation (42). Under normal physiological conditions, HDL-C can regulate the inflammatory response through multiple mechanisms and maintain the immune balance in the body. However, when a pathological state occurs in the bladder, such as in the case of OAB, the inflammatory microenvironment in the body may interfere with the normal metabolism and function of HDL-C. On the one hand, in a chronic inflammatory state, the synthesis of ApoA1 in the liver decreases, and at the same time, the serum immunoglobulin A (sAA) increases, leading to a decrease in the level of HDL-C (43). On the other hand, the anti-oxidative and anti-inflammatory activities of HDL-C may be weakened in an inflammatory state (44). In this situation, the change in the level of HDL-C may reflect the impact of the systemic inflammatory state caused by bladder-related diseases on lipid metabolism and immune regulation. Therefore, the change in the level of HDL-C in NHR can also, to a certain extent, reflect the pathological processes related to the bladder. In addition, considering the perspective of the neuro-endocrine-immune regulatory network, the function of the bladder is jointly regulated by the nervous, endocrine, and immune systems (45). The systemic inflammatory state may interfere with the balance of this regulatory network and affect the normal physiological function of the bladder. As an indicator reflecting the systemic inflammatory and lipid metabolism states, the change in NHR may be related to the imbalance of the neuro-endocrine-immune regulatory network. For example, inflammatory factors may interfere with the neural regulation of the bladder by affecting the synthesis and release of neurotransmitters, leading to the occurrence of symptoms such as overactive bladder (46, 47).

Regarding the nonlinear trend, we propose the following hypothetical explanations, which are yet to be further verified by subsequent research. Firstly, although HDL-C has an anti-inflammatory effect, there may be a potential threshold effect in the realization of its function. As shown in the study by Yuksel et al. (33), an HDL-C level of less than 60 mg/dL is a risk factor for OAB. This suggests that when the HDL-C level is low, its impact on OAB may be more significant, and the pattern of this impact may change after exceeding a certain threshold. In addition, although all white blood cells are involved in the body’s anti-infection process, neutrophils play a crucial role in defending against certain infections, especially bacterial infections. An increasing amount of evidence indicates that chronic low-grade bacterial bladder colonization may exacerbate the symptoms of OAB and may explain why the current treatment strategies for OAB are not always successful (48). The chemotaxis of neutrophils towards the infection site depends on the binding of CXCL-8 to the G protein-coupled cell surface receptors CXCR1 and CXCR2 expressed by infected epithelial cells. After binding, it can exert anti-inflammatory and antibacterial effects (49, 50). Thus, it is speculated that an insufficient number of neutrophils may be one of the risk factors for OAB. Based on the above analysis, we hypothesize that these mechanisms may explain why, when the NHR is low, the risk of developing OAB shows a downward trend as the NHR increases. However, this explanation is currently only a hypothetical speculation based on existing research, and its specific mechanisms and causal relationships still need to be deeply verified in the future through various research methods such as large-sample prospective studies and basic experiments.

From a clinical perspective, the association observed between NHR and OAB in this study has certain potential clinical relevance. Firstly, as an easily obtainable inflammatory biomarker, NHR can serve as one of the indicators for the preliminary assessment of the risk of developing OAB during clinical diagnosis and treatment. When clinically evaluating suspected OAB patients, by measuring the patients’ NHR levels and combining them with other clinical symptoms and examination results, a more comprehensive assessment of the patients’ conditions can be achieved. For example, for patients with a high NHR level, clinicians can be more vigilant about the possibility of them having OAB and conduct further detailed urological examinations, such as urodynamic tests and bladder ultrasound, in order to detect and diagnose OAB at an early stage and secure a more timely treatment opportunity for the patients. Secondly, from the perspective of treatment, if future studies further confirm the causal relationship between NHR and OAB, then intervening in the inflammatory and lipid metabolism abnormalities reflected by NHR may provide new strategies for the treatment of OAB. For instance, by regulating the patients’ lipid metabolism to increase the HDL-C level, or controlling the inflammatory response to reduce the number of neutrophils, it may be possible to improve the symptoms of OAB patients and enhance their quality of life. This may involve adjusting the patients’ lifestyle, such as dietary control and increased physical activity, as well as using medications for intervention when necessary. In addition, for patients who have already been diagnosed with OAB, monitoring the changes in NHR levels may help evaluate the treatment effect and the progression of the disease. If the patients’ NHR levels gradually decrease during the treatment process, it may suggest that the treatment measures are effective in improving the patients’ inflammatory and metabolic status, and thus have a positive impact on the relief of OAB symptoms. Conversely, if the NHR levels continue to rise, it may be necessary to re-evaluate the treatment plan and adjust the treatment strategy.

NHR is an inflammatory biomarker that is not only associated with OAB. Some other diseases, such as cancer, along with CVD and metabolic disorders, are also related to NHR (51–53). When distinguishing different diseases associated with NHR, comprehensive judgments can be made from multiple aspects. In terms of clinical symptoms and signs, OAB is mainly characterized by storage-phase symptoms such as urinary urgency, frequency, and nocturia (5). CVD presents with manifestations such as chest pain and dyspnea, and metabolic disorders like diabetes have features such as polydipsia, polyphagia, and polyuria (54). Regarding auxiliary examinations, for CVD, it is necessary to pay attention to myocardial injury markers and lipid parameters, and combine imaging examinations such as electrocardiogram and echocardiogram (55). OAB, on the other hand, relies on urinary system ultrasound and urodynamic tests, and metabolic disorders have specific laboratory indicators and targeted imaging examinations (56). For example, vitamin D deficiency in healthy and metabolically normal women can be used in combination with the monocyte-to-high-density lipoprotein ratio (MHR) to aid in differential diagnosis (57). In addition, medical history and family history are also of great reference value. CVD and metabolic disorders often have a family genetic predisposition, while OAB is mostly related to local abnormalities of the urinary system or neurological system. By integrating this information, various diseases can be effectively distinguished, providing a basis for diagnosis and treatment (2, 58). Finally, regarding cancer-related diseases, apart from specific imaging findings and targets, an elevated level of MASLD and fasting plasma glucose (FPG) may play a role in the clinical context of colorectal cancer (CRC) (59).

### 4.1 Strengths and limitations

This study mainly has three advantages. First, we include 29,315 participants from seven rounds of the National Health and Nutrition Examination Survey (2005 - 2018), and after weighting, it represents approximately 180 million non-hospitalized American adults, which makes our research results generally representative. Second, detailed subgroup analysis and adjustment of key covariates enhance the robustness of this study. Finally, this study also conducts nonlinear analysis and piecewise regression on the relationship between NHR and OAB. This analysis goes beyond the simple association in the traditional sense.

However, certain limitations should be acknowledged. First, limited by the cross-sectional design type, this study cannot determine the causal relationship between NHR and OAB. To address this, future research could adopt a prospective cohort study design. By following a large group of participants over an extended period, researchers can monitor the development of OAB symptoms and changes in NHR values, thereby establishing temporal relationships and inferring causal associations. Additionally, incorporating randomized controlled trials, if feasible, could further validate the causal link by manipulating specific variables in a controlled environment. Second, the values of neutrophils and HDL-C may be biased because the acquisition of these data may be affected by multiple factors such as the environment and psychological state at the time of detection. To mitigate this issue, future studies should standardize the data collection process. This could involve specifying strict criteria for the time of day, patient fasting status, and pre-collection activities for biomarker measurements. Moreover, integrating wearable devices or continuous monitoring technologies to record real-time physiological and environmental data may help identify and adjust for confounding factors that influence biomarker levels. In addition, the diagnosis of OAB may not be particularly accurate because the NHANES data set lacks laboratory tests, and the diagnosis of OAB completely depends on OABSS. In future research, combining self-reported OABSS scores with objective diagnostic methods, such as urodynamic testing, urine flow rate measurements, and bladder ultrasound, can enhance the accuracy of OAB diagnosis. Collaborating with clinical institutions to collect additional diagnostic data from participants would provide a more comprehensive assessment of OAB status. Finally, collecting symptoms through questionnaires is susceptible to recall bias. To reduce recall bias, future studies can utilize electronic health records (EHRs) to obtain objective medical history data, which are less prone to memory errors. Additionally, employing repeated questionnaires at regular intervals during the study period can help cross-reference information and minimize recall inaccuracies. Training interviewers to use standardized questioning techniques and providing clear guidelines to participants may also improve the reliability of self-reported data.

## 5 Conclusion

This study suggests a non-linear association between NHR and OAB. When NHR ≥ 2.85, there is a strong positive association between NHR and the odds of OAB. The interaction of obesity and NHR further increases the prevalence of OAB. Further prospective, multicenter studies are needed to clarify the underlying mechanisms and determine the causal relationship between NHR and OAB.

## Data Availability

Publicly available datasets were analyzed in this study. This data can be found here: The data used in this study are available at https://wwwn.cdc.gov/nchs/nhanes/.
