# Supplementary material for: Association of neutrophil to high-density lipoprotein cholesterol ratio with overactive bladder: a population-based study
Source: Front Endocrinol (Lausanne). 2025 May 22;16:1541294. doi: 10.3389/fendo.2025.1541294 (PMC12137064; doi:10.3389/fendo.2025.1541294)
Supplement: Supplementary file 1 [file Table1.docx]

**Association of neutrophil to high-density lipoprotein cholesterol ratio (NHR) with Overactive Bladder (OAB): a population-based study**

## Supplementary Figure 1. Flow diagram of the overactive bladder diagnosis based on overactive bladder syndrome score(1).


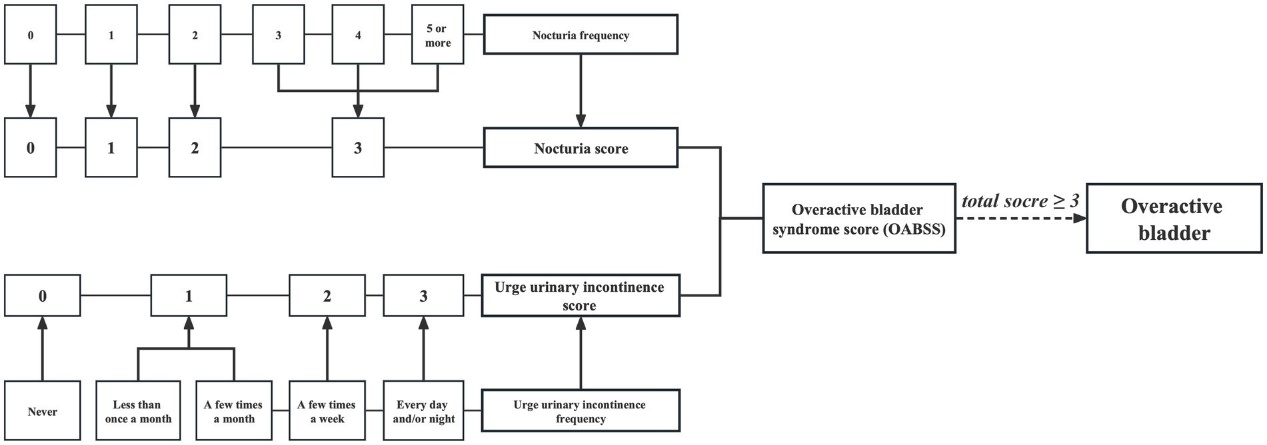


1. Zhang Y, Song J, Li B, Wu Y, Jia S, Shu H, et al. Association between body roundness index and overactive bladder: results from the NHANES 2005-2018. *Lipids Health Dis* (2024) 23(1):184. doi: 10.1186/s12944-024-02174-1

## Supplementary Table 1. Association between NHR and OAB in NHANES 2005-2020

|  | Model 0 | | Model 1 | | Model 2 | |
| --- | --- | --- | --- | --- | --- | --- |
|  | OR (95%CI) | *P-*value | OR (95%CI) | *P-*value | OR (95%CI) | *P-*value |
| per unit | 1.04(1.02,1.07) | <0.0001 | 1.07(1.04,1.10) | <0.0001 | 1.05(1.02,1.08) | <0.001 |
| Quartile 1 | ref |  | ref |  | ref |  |
| Quartile 2 | 0.96(0.85,1.08) | 0.46 | 1.01(0.88,1.15) | 0.92 | 0.97(0.86,1.11) | 0.69 |
| Quartile 3 | 1.06(0.93,1.20) | 0.38 | 1.15(1.00,1.32) | 0.05 | 1.08(0.94,1.24) | 0.28 |
| Quartile 4 | 1.16(1.04,1.30) | 0.01 | 1.34(1.18,1.52) | <0.0001 | 1.20(1.06,1.35) | 0.004 |
| *P* for trend |  | 0.002 |  | <0.0001 |  | 0.001 |

Notes: Model 0 did not adjust for any confounding factors; Model 1: Adjust for age, sex, race/ethnicity, marital status, poverty income ratio, educational level, drinking status, smoking status, healthy diet, physical activity, and body mass index. Model 2 further adjusts for cancer, diabetes, hypertension, chronic kidney disease, cardiovascular disease, liver disease, hyperlipidemia, and use of lipid-lowering drugs based on Model 1. OR odds ratio, CI confidence interval, OAB overactive bladder, NHR neutrophil to high-density lipoprotein cholesterol ratio, NHANES National Health and Nutrition Examination Survey.

## Supplementary Table 2. Association between NHR and OAB through segmented NHR in NHANES 2005-2020.

|  | Model 0 | | Model 1 | | Model 2 | |
| --- | --- | --- | --- | --- | --- | --- |
|  | OR (95%CI) | *P-*value | OR (95%CI) | *P-*value | OR (95%CI) | *P-*value |
| *<2.85* | 0.90(0.81,1.00) | 0.04 | 0.93(0.82,1.05) | 0.23 | 0.91(0.80,1.03) | 0.12 |
| *>=2.85* | 1.05(1.02,1.08) | <0.001 | 1.07(1.04,1.11) | <0.001 | 1.05(1.01,1.09) | 0.01 |

Notes: Model 0 did not adjust for any confounding factors; Model 1: Adjust for age, sex, race/ethnicity, marital status, poverty income ratio, educational level, drinking status, smoking status, healthy diet, physical activity, and body mass index. Model 2 further adjusts for cancer, diabetes, hypertension, chronic kidney disease, cardiovascular disease, liver disease, hyperlipidemia, and use of lipid-lowering drugs based on Model 1. OAB overactive bladder, NHR neutrophil to high-density lipoprotein cholesterol ratio, OR odds ratio, CI confidence interval, NHANES National Health and Nutrition Examination Survey.
